# Machine-Learning-Based Prediction of Clinical Outcomes in Gliomas Using Glycomic Features

**DOI:** 10.34133/csbj.0153

**Published:** 2026-07-09

**Authors:** Xin Ma, Derek Allison, Jessica K.A. Macedo, Kayli E. Bolton, Harrison Clarke, Lyndsay E.A. Young, Jiang Bian, Janna H. Neltner, Craig Vander Kooi, Ramon C. Sun, Matthew S. Gentry, Li Chen

**Affiliations:** ^1^ Department of Biostatistics, College of Public Health and Health Professions and College of Medicine, University of Florida, Gainesville, FL, USA.; ^2^ Center for Advanced Spatial Biomolecule Research, University of Florida, Gainesville, FL, USA.; ^3^ Department of Pathology and Laboratory Medicine, College of Medicine, University of Kentucky, Lexington, KY, USA.; ^4^ Markey Cancer Center, University of Kentucky, Lexington, KY, USA.; ^5^ Department of Molecular and Cellular Biochemistry, College of Medicine, University of Kentucky, Lexington, KY, USA.; ^6^ Department of Biochemistry and Molecular Biology, College of Medicine, University of Florida, Gainesville, FL, USA.; 7 Department of Pharmacology and Immunology and Hollings Cancer Center, Medical University of South Carolina, Charleston, SC, USA.; ^8^ Regenstrief Institute, Indianapolis, IN, USA.; ^9^ Biostatistics and Health Data Science, School of Medicine, Indiana University, Indianapolis, IN, USA.; ^10^ McKnight Brain Institute, University of Florida, Gainesville, FL, USA.

## Abstract

**Background:** Gliomas are primary malignant brain tumors. Among them, grade IV astrocytomas represent the most aggressive form with limited treatment options and a poor prognosis. There is a critical need for reliable prognostic biomarkers to predict patient outcomes. **Methods:** In this study, we evaluated the prognostic roles of glycomics in predicting outcomes of patients with glioma. To address this need, we collected a cohort of 86 patients and conducted N-linked glycomics analysis using matrix-assisted laser desorption/ionization mass spectrometry on a tissue microarray comprising 78 gliomas and 8 controls. We performed survival analyses and machine-learning-based predictive modeling to evaluate the prognostic roles of glycomic features. **Results and Conclusion:** Glycomic features alone displayed strong performance in classifying mortality status, achieving a mean area under the receiver operating characteristic curve (AUROC) value of 0.880 and a mean area under the precision–recall curve (AUPRC) value of 0.925, and demonstrating consistent prognostic value in survival analyses with a mean concordance index of 0.759 across repeated cross-validation. Glycomic features also showed strong performance in predicting the incidence of seizure status, with a mean AUROC value of 0.778 and a mean AUPRC value of 0.766. Additionally, top-ranked glycans from machine-learning models can effectively stratify patients based on their survival outcomes, demonstrating their potential role as prognostic biomarkers.

## Introduction

Gliomas are primary brain tumors arising from glial cells with varying degrees of malignancy. Among them, grade IV astrocytomas are the most common and aggressive primary brain tumor in adults, marked by rapid progression, extensive invasion into surrounding brain tissue, and a high degree of resistance to current therapies [1]. Even with aggressive treatment strategies, including surgical resection, radiation therapy, and chemotherapy with temozolomide, the median survival for patients with grade IV astrocytoma remains dismal, ranging from 12 to 15 months, with a 5-year survival rate of less than 10% [2]. The severity of grade IV astrocytomas is largely attributed to its genetic heterogeneity, diffuse infiltration, and the ability to evade conventional treatments, leading to high recurrence rates. Efforts to improve outcomes have focused on identifying molecular biomarkers, such as mutations in *IDH1* and *TP53*, *EGFR* amplification, and *MGMT* promoter methylation, which are associated with treatment response and overall survival [3,4]. While these markers have shown promise in guiding therapeutic decisions, they are insufficient for reliably predicting patient prognosis, particularly in cases without these specific mutations. Therefore, the need for novel, more robust prognostic biomarkers that can accurately predict patient survival regardless of therapeutic interventions remains a critical challenge. Identifying such biomarkers could enable better patient stratification for more personalized management approaches and ultimately improve survival outcomes in this highly aggressive cancer.

Machine learning (ML) and artificial intelligence (AI) have emerged as powerful tools in cancer biomarker discovery, particularly for predicting patient outcomes and identifying prognostic biomarkers [5]. ML/AI algorithms enable the integration of multidimensional data sources, leading to more comprehensive and accurate models of disease progression and patient prognosis. By analyzing large and complex datasets, ML/AI algorithms can also uncover intricate patterns and relationships across genomic, proteomic, metabolomic, and clinical data that traditional statistical methods might overlook [6]. In oncology, ML/AI have facilitated the development of predictive models for various cancers, enhancing the precision of survival predictions and informing personalized treatment strategies [7]. In the context of gliomas, ML/AI has been applied to integrate diverse data types, such as clinical features, genomic mutations, gene expression profiles, epigenetic modifications, and imaging features, for survival analysis and outcome prediction [8]. For example, clinical features derived from the national Surveillance, Epidemiology, and End Results Program database have been used in several ML algorithms, such as XGBoost, random forest, and deep neural network, to predict the survival outcomes of patients with glioblastoma [9]. MRI radiomics in conjunction with molecular profiles have been incorporated in a joint learning ML approach for discovering glioblastoma subtypes [10]. These applications highlight the important potential of ML/AI to enhance prognostic biomarker discovery in gliomas, offering more precise tools for clinical decision-making and personalized patient management. However, despite these advancements, challenges remain in integrating less-explored molecular features, such as glycomics, into prognostic models. Glycomics is the study of complex carbohydrates that includes glycogen, O-linked glycosylation, and N-linked glycosylation. N-linked glycans are branched oligosaccharides that are added co- and posttranslationally to asparagine residues of proteins in the endoplasmic reticulum and Golgi. These sugar modifications impact protein folding, stability, trafficking, and receptor–ligand interactions at the cell surface. In the brain, N-linked glycosylation is critical for synaptogenesis, plasticity, axon guidance, myelination, and neurotransmitter receptor trafficking to maintain excitatory/inhibitory balance. Dysregulation of N-linked glycans perturbs growth factors, adhesion, and ion-channel function, thus this emerging field holds promise for uncovering new biomarkers in cancer progression and metastasis, especially in brain cancers [11–12]. Incorporating N-linked glycan profiles into ML/AI algorithms could reveal novel prognostic markers and further improve the accuracy of survival predictions by integrating glycomics with clinical and other molecular data. Such an approach could lead to more robust predictive models that account for the multifaceted nature of the disease, enabling more accurate outcome predictions, personalized treatments, and improved gliomas survival rates. Recently, glycomics has been leveraged by ML approaches to assess chemotherapy response in patients with lung cancer, predict gastric cancer risk, and diagnose multiple cancers including ovarian cancer, non-small cell lung cancer, gastric cancer, and esophageal cancer [13–15]. Nevertheless, despite the potential crucial role glycans may play in gliomas, the study of glycans in patients with glioma from a population perspective using ML approaches is underexplored. To fill the gap, in this work, we analyzed tissue microarrays (TMAs) comprising tumor specimens from a cohort of 78 patients with glioma at different cancer grades along with 8 controls and performed matrix-assisted laser desorption/ionization (MALDI) mass spectrometry to obtain their glycan profiles. We further leveraged N-glycan features to predict clinical outcomes in patients with glioma and identify key glycans associated with prognosis. The prediction performance and identified cancer-associated glycans highlight the potential role of N-glycan for gliomas.

## Materials and Methods

### Glioma cohort

Eighty-six specimens (males *n* = 55; females *n* = 31) from a cohort of patients undergoing resective surgery were reviewed by board-certified neuropathologists. Tumor samples were classified according to World Health Organization grading criteria at the time of pathology review. For TMA construction, representative tumor-rich regions (>70% tumor cellularity) were selected to maximize lesional tissue and minimize necrosis or nontumor brain. All specimens were collected at the University of Kentucky HealthCare and stored by the Biospecimen Procurement and Translation Pathology Shared Resource Facility of the Markey Cancer Center under an approved institutional review board. The samples were coupled with de-identified demographic and clinical data and consisted of 78 specimens with brain tumors and 8 specimens with cortical dysplasia that were used as a control. Demographic and clinical information is shown in Table 1. The samples were then used to generate TMAs except 2 brain tumors in Grade I. For data summary and survival analysis based on cancer grade, we used all 86 samples. For other analyses, we used 84 samples profiled with 62 usable glycans.

**Table 1.** Distribution of age at surgery, gender, seizure, mortality status, and survival time for patients with glioma in control and each cancer grade (Grade I to Grade IV)

Characteristic	Control *N* = 8 ^a^	Grade I *N* = 2 ^a^	Grade II *N* = 25 ^a^	Grade III *N* = 14 ^a^	Grade IV *N* = 37 ^a^	Overall *N* = 86 ^a^	*P* value ^b^
Age at surgery	36.8 (10.30)	16.5 (1.50)	36.6 (12.61)	45.3 (11.06)	56.8 (16.47)	46.3 (17.08)	<0.001
Sex							0.5
F	1 (13%)	1 (50%)	8 (32%)	7 (50%)	14 (38%)	31 (36%)	
M	7 (88%)	1 (50%)	17 (68%)	7 (50%)	23 (62%)	55 (64%)	
Seizure history							0.016
Epilepsy	7 (88%)	0 (NA%)	6 (33%)	1 (14%)	2 (25%)	16 (39%)	
Seizure	1 (13%)	0 (NA%)	12 (67%)	6 (86%)	6 (75%)	25 (61%)	
Unknown	0	2	7	7	29	45	
Died	0 (0%)	0 (0%)	5 (20%)	8 (57%)	36 (97%)	49 (57%)	<0.001
Overall survival (mo)	53.6 (34.68)	80.4 (14.36)	64.8 (33.67)	45.4 (38.24)	14.4 (15.56)	39.3 (35.70)	<0.001

^a^ Mean (SD); *n* (%).

^b^ Kruskal–Wallis rank-sum test; Fisher exact test.

### TMA production and enzyme digestion

Formalin-fixed paraffin-embedded glioma TMA slides were processed as previously described [16–19]. In brief, tissues were dewaxed and rehydrated followed by antigen retrieval. Recombinant PNGase F (0.1 μg/μl) and isoamylase (Megazyme 3 U) dialyzed into ultrapure water were applied using an M5 Sprayer Robot (HTX Technologies LLC, Chapel Hill, NC). Enzymes were sprayed onto the slide at a rate of 25 μl/min with a 0-mm offset and a velocity of 1,200 mm/min at 45 °C and 10 psi for 15 passes, followed by a 2-h incubation at 37 °C in a prewarmed humidity chamber. After incubation, slides were desiccated for 15 min and 7 mg/ml alpha-cyano-4-hydroxycinnamic acid matrix in 50% acetonitrile with 0.1% trifluoroacetic acid was applied using the M5 Sprayer at 0.1 ml/min with a 2.5-mm offset and a velocity of 1,300 mm/min at 79 °C and 10 psi for 10 passes. Slides were stored in a desiccator or immediately used for MALDI–mass spectrometry imaging (MSI) analysis.

### Glycomics MALDI–MSI analysis

MSI analysis was conducted as previously described [20,21] with a few modifications. Briefly, a Synapt G2Si mass spectrometer (Waters Corporation, Milford, MA) equipped with an Nd:YAG UV laser operating at 1,000 Hz, energy of 200 AU, and spot size of 50 μm was used to detect released N-glycans at *X* and *Y* coordinates of 100 μm. The mass range was preset to 500 to 3,500 *m*/*z*. Following data acquisition, spectra were uploaded to High-Definition Imaging (HDI) Software (Waters Corporation) for mass range analysis. For N-glycan quantification, regions of interest were defined for each patient sample and for all pixels obtained within a region of interest, peak intensities were averaged and normalized by total ion current. Peak intensities were also normalized by the averaged intensity from both TMAs combined. HDI-generated glycan images were obtained for the most abundant N-glycans detected across all patient samples. Representative glycan structures were generated in GlycoWorkbench.

### Statistical analysis

All statistical analyses were performed using R (version 4.3.3). For exploratory data analysis (Table 1), for continuous variables, such as age at surgery and survival time in months, mean and standard deviation were calculated and were assessed for statistical significance using Kruskal–Wallis rank-sum test. For categorical variables, including gender, seizure status, and mortality status, frequency and column percentage were calculated and were analyzed using Fisher exact test to evaluate associations across different levels. Survival analysis and Kaplan–Meier (KM) curves plot was performed using R package “survival” (version 3.8.3).

### Machine-learning predictive modeling

Four machine-learning models have been performed in the study, which include Random Forest (RF), implemented using R package “randomForest” (version 4.7.2); Lasso, implemented using R package “glmnet” (version 4.1.10); Support Vector Machine (SVM), implemented using R package “e1071” (version 1.7.16); and Random Survival Forest, implemented in R package “randomForestSRC” (version 3.5.1). The model training and testing process used 10 repetitions of 5-fold cross-validation approach to generate area under the receiver operating characteristic curve (AUROC) and area under the precision–recall curve (AUPRC) values for binary prediction. In each repetition, the full dataset was split into 5 folds, where each fold served as a test set once, while the remaining 4 folds were used for training. This iterative process generated 5 sets of out-of-sample predictions per repetition. For each fold, AUROC and AUPRC were computed on the test set, resulting in a total of 50 evaluations across all repetitions. The final performance was obtained by averaging these metrics across all folds and repetitions. For binary prediction, model selection was first performed among 3 machine-learning models (RF, Lasso, and SVM), each using the same set of clinical predictors including cancer grade, age at surgery, and gender. Following model selection, additional comparisons were conducted using the RF model to evaluate the impact of different predictor combinations including clinical variables only and glycomic features only. This analysis aimed to assess whether glycomic features could achieve predictive accuracy comparable to that of clinical variables. For predicting time-to-event outcome, Random Survival Forest was applied using the same feature set (i.e., clinical variables only and glycomic features only) under the same cross-validation strategy, and model performance was evaluated using the concordance index (C-index). We identified top-ranked glycomic features from the RF or Random Survival Forest models using permutation importance, which quantifies the feature importance as the reduction in prediction metric after randomly permutating its values in the out-of-bag samples. Features that led to a greater decrease in prediction metric were considered more important. KM survival curves were generated for the most influential glycomic features to examine their effect on survival probability. The log-rank test was used to compare KM curves across quartiles based on glycan abundance, with *P* values indicating the statistical significance of survival differences.

## Results

### Patient cohort and TMA construction

We constructed a TMA comprising tumor specimens from a cohort of 86 patients diagnosed with gliomas. The patient cohort included individuals with varying tumor grades, seizure status, age, sex, and time of death, reflecting the diverse clinical presentations of gliomas (Table 1). The primary objective of constructing this TMA was to facilitate the study of molecular features associated with gliomas and to identify trends related to disease progression and patient outcomes. The TMA was curated by neuropathologists who reviewed each tumor sample to ensure the accuracy and quality of the tissue cores. For each case, areas containing more than 70% tumor cells were identified and selected for inclusion in the TMA. This selection process aimed to maximize the representation of tumor tissue while minimizing nontumor elements, such as necrosis or normal brain tissue. By focusing on regions with high tumor cell density, the TMA provided a robust platform for subsequent molecular analyses, enhancing the reliability of detecting potential biomarkers associated with glioma progression and patient survival. Table 1 summarizes the demographic and clinical characteristics of the patient cohort (*n* = 86) stratified by cancer grade, including control, Grade I, Grade II, Grade III, and Grade IV groups. Significant differences in age at surgery were observed across groups (*P* < 0.001), with the mean age increasing progressively from Grade I to Grade IV. Gender distribution did not differ significantly between groups (*P* = 0.5). Seizure and mortality status showed significant associations with cancer grade (*P* = 0.016 and *P* < 0.001, respectively), with a higher prevalence of seizure and mortality status in higher-grade cancers. Median survival time (months) also decreased with advancing cancer grade, demonstrating a significant difference (*P* < 0.001).

**Fig. 1.**
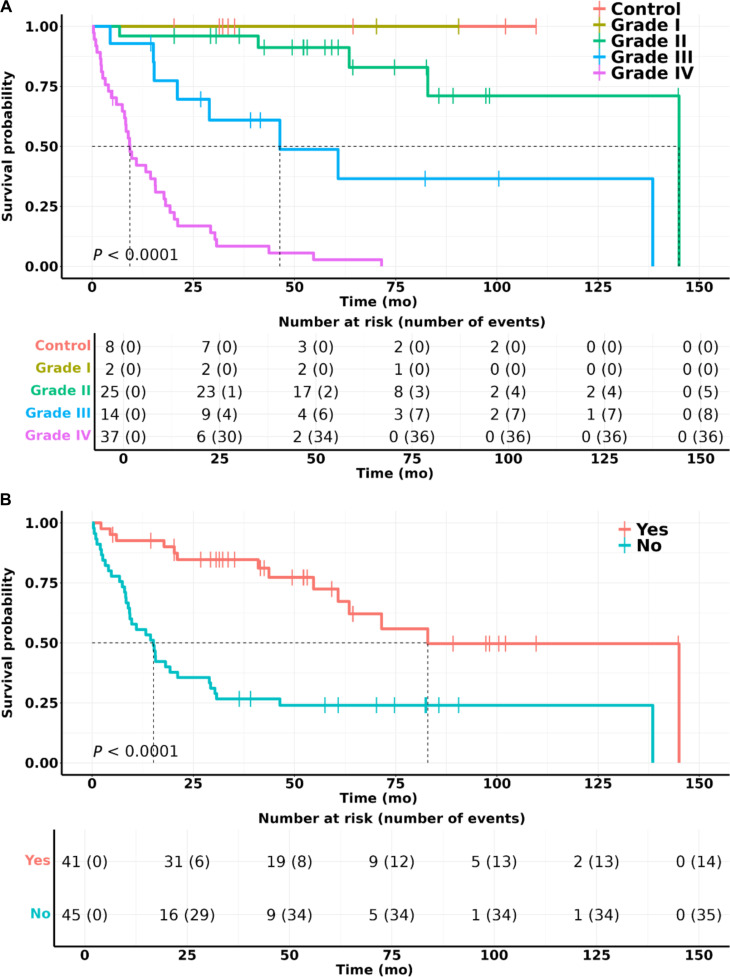
Kaplan–Meier (KM) survival analyses by cancer grade and seizure status. (A) Stratified by control and 4 cancer grades. (B) Stratified by seizure status (yes or no).

To ensure that our TMA cohort accurately represents the typical distribution of clinical characteristics in patients with glioma, we performed KM survival analyses to assess the impact of cancer grade and seizure status on survival probability (Fig. 1). As expected, the KM survival curves revealed that patients with higher cancer grades (III and IV) had significantly lower survival rates compared to control and lower-grade groups (*P* < 0.0001), as illustrated in Fig. 1A. Furthermore, Fig. 1B demonstrates a significant difference in survival probability between patients with and without seizure status (*P* < 0.0001). Interestingly, the presence of seizure status is associated with better survival outcomes, consistent with previous findings that seizure is correlated with better survival outcomes in brain tumors such as gliomas [22,23]. These results align with established clinical observations in patients with these tumors, confirming that our TMA cohort reflects a representative distribution of prognostic factors. This validation supports the suitability of our cohort for subsequent molecular analyses aimed at identifying biomarkers related to disease progression and patient survival.

### Glycomic profiling of the tissue microarray using MALDI mass spectrometry

**Fig. 2.**
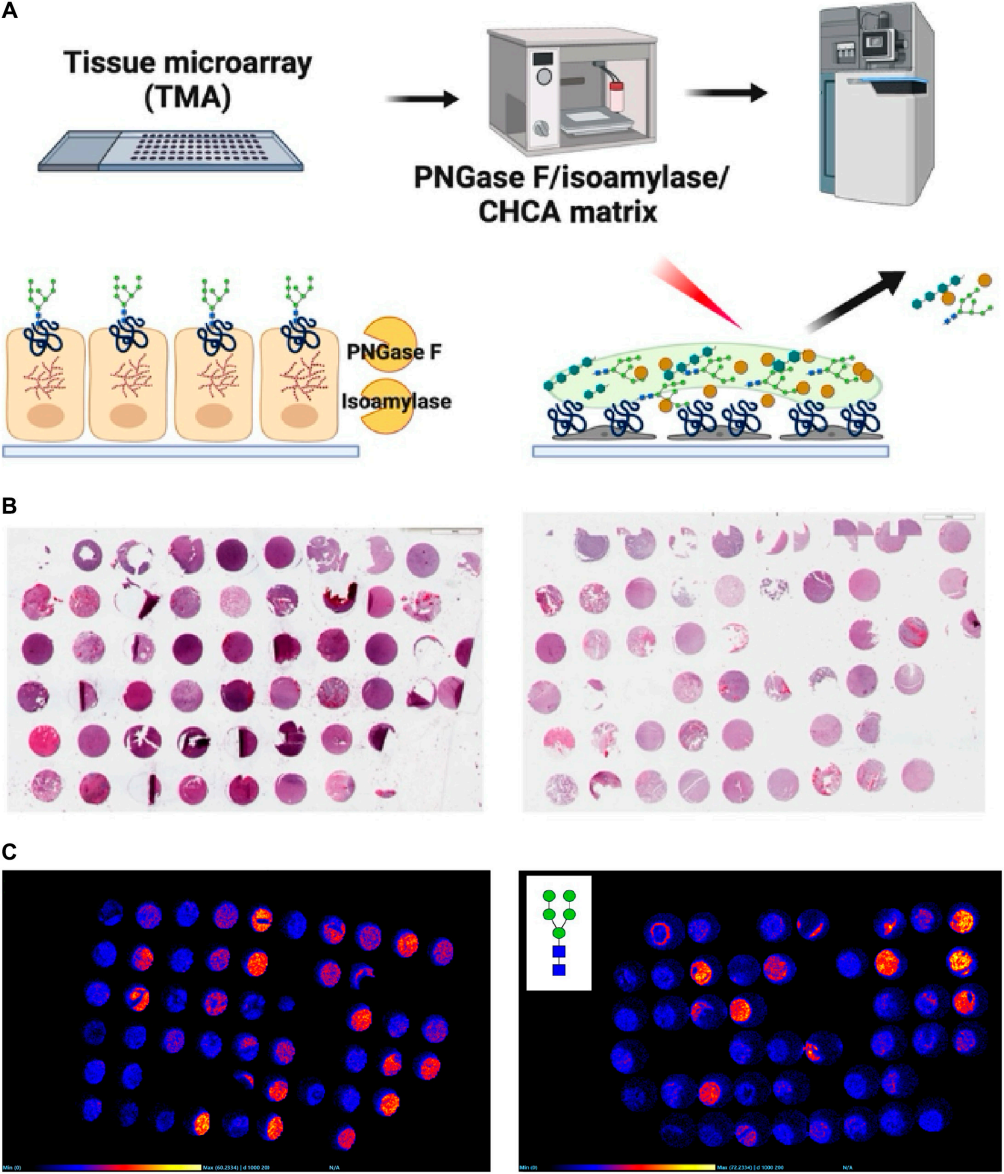
(A) The workflow of matrix-assisted laser desorption/ionization (MALDI) mass spectrometry on tissue microarray (TMA). (B) Hematoxylin-and-eosin (H&E) images of the adjacent sections of TMA scanned by MALDI. (C) The pseudo-color scale, assigning gradually changing colors to the intensity ranges from blue (lowest intensity) to red to yellow (highest intensity).

Using the constructed TMA, we performed tissue glycomics analysis employing MALDI mass spectrometry based on previously established techniques [16–19]. The enzyme peptide *N*-glycosidase F (PNGase F) was applied to the tissue cores using a highly efficient and uniform sprayer, ensuring consistent enzyme distribution across all samples. The TMA was then incubated at 37 °C to facilitate the release of protein-bound N-linked glycans from the tissue sections. Following enzymatic digestion, a suitable matrix was applied to the TMA in preparation for MALDI mass spectrometry analysis. This approach yielded strong and consistent glycan signals from the TMA, indicating successful extraction and detection of N-linked glycans from the tissue samples. The high-quality glycomic data obtained provided a robust basis for subsequent analyses exploring the relationship between glycan profiles and clinical parameters in patients with glioma (Fig. 2).

### Initial glycomic analysis and correlation with cancer grade

**Fig. 3.**
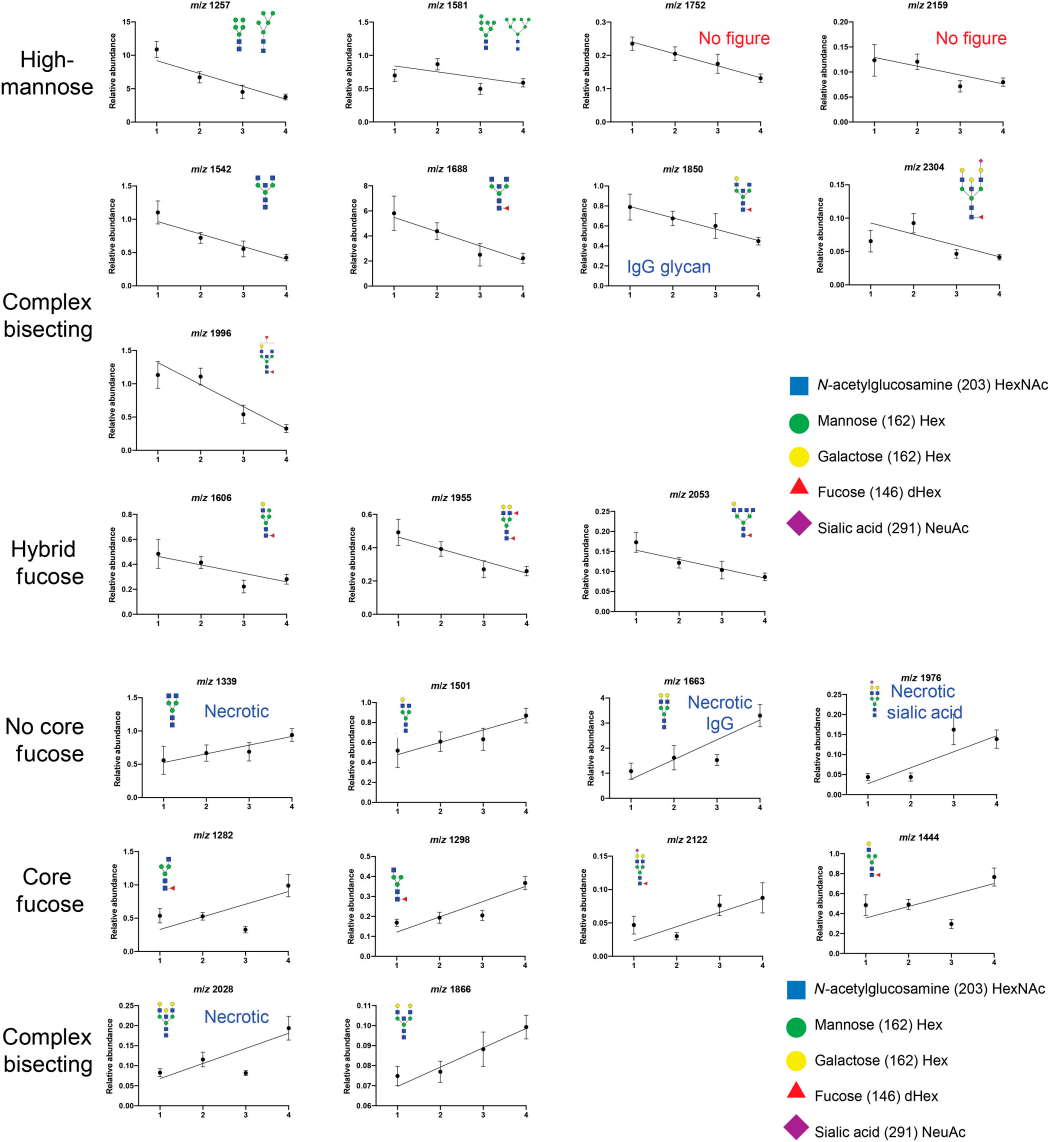
Examples of glycans with strong positive or negative correlation between expression level and cancer grade.

In our initial analysis of the glycomic data obtained from the TMA, we identified several glycans whose abundance levels correlated significantly with cancer grade, exhibiting both positive and negative associations (Fig. 3). Specifically, we observed that certain high-mannose glycans were uniquely expressed or upregulated in higher-grade tumors, suggesting a potential link to increased tumor aggressiveness. Additionally, core-fucosylated glycans showed notable correlations, with some species being more abundant in advanced cancer grades. Complex bisecting N-glycans were also found to correlate with cancer grade, displaying varied abundance patterns that may reflect alterations in glycosylation pathways associated with tumor progression. These findings indicate that specific glycan structures, including unique high-mannose, core-fucosylated, and complex bisecting glycans, are differentially expressed across glioma grades and may serve as potential biomarkers for tumor grading and prognostication.

### Application of machine learning for prognosis prediction using clinical variables and glycomic features

**Fig. 4.**
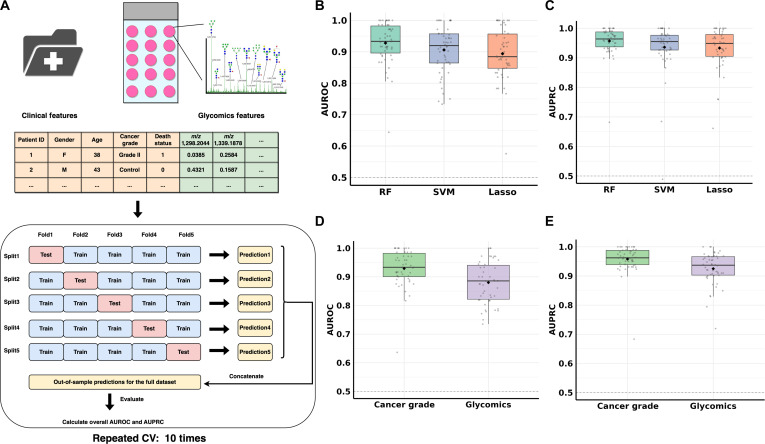
Predicting mortality status using cancer grade or glycomic features. (A) The data structure of patient outcome, clinical features and glycomic features, and evaluating strategy using 10 repeated 5-fold cross-validation (CV). (B) Area under the receiver operating characteristic curve (AUROC) reported by 3 machine-learning models solely using cancer grade, adjusted for gender and age at surgery. (C) Area under the precision–recall curve (AUPRC) reported by 3 machine-learning models solely using cancer grade, adjusted for gender and age at surgery. (D) AUROC reported by Random Forest using cancer grade or glycomics, adjusted for gender and age at surgery. (E) AUPRC reported by Random Forest using cancer grade or glycomics, adjusted for gender and age at surgery.

Building on the promising results from our initial glycomic analysis, we explored the potential of ML algorithms for glioma prognosis using glycomic features. Recognizing that specific glycan abundance correlated with cancer grade (Table 1 Fig. 3), we aimed to assess whether glycomics could provide prognostic insights comparable to cancer grade. These observations provide insights for the role of N-glycans as potential alternative or complementary sources of prognostic information. We tested the hypothesis by employing 3 popular ML methods, namely, RF, SVM, and Lasso, to classify mortality status. We first chose the best ML method by using solely clinical features, primarily cancer grade, adjusted for age at surgery and gender, through 10 repeated 5-fold cross-validation (Fig. 4A). The performance of each model was evaluated using the AUROC and the AUPRC. As illustrated in Fig. 4B and C and Table S1, RF outperformed the other models, achieving the highest mean AUROC value of 0.928 and AUPRC value of 0.957. In comparison, Lasso attained a mean AUROC value of 0.894 and a mean AUPRC value of 0.933, while SVM achieved a slightly higher mean AUROC value of 0.906 and mean AUPRC value of 0.936. These results indicate that RF was the most effective model for predicting mortality status based on clinical features. Consequently, RF was selected as the model for subsequent mortality-status classification analyses.

Further investigation using RF focused on comparing the prognostic power of glycomic features and cancer grade for predicting mortality status adjusted for age at surgery and gender. Figure 4D and E and Table S2 illustrate the AUROC and AUPRC values for models based on glycomics and cancer grade. We observed that using solely glycomic features can achieve promising prediction accuracy with a mean AUROC value of 0.880 and mean AUPRC value of 0.925. This performance was slightly lower than that of the models using cancer grade only (AUROC = 0.929 and AUPRC = 0.958). These results indicate that glycomics possess prognostic capability approaching the performance of cancer grade, highlighting their potential as valuable indicators of patient outcomes. To identify the features contributing most to the prediction, we extracted feature importance from the trained RF model using glycomic features. Notably, several glycomic markers, including Glycan 1996, Glycan 1542, and Glycan 1257, exhibited high importance scores, indicating their strong predictive value for mortality status. Additionally, age at surgery also contributed substantially to model performance, while gender plays a minor role in the prediction (Fig. S1). Similarly, RF outperforms SVM and Lasso in predicting seizure status by obtaining higher AUROC and AUPRC values (Fig. S2AB and Table S3). Additionally, using glycomics alone achieved prediction performance, which approached that of cancer grade (Fig. S2CD and Table S4). Glycan 1996 and Glycan 1257 are also top-ranked important features, indicating their prognostic roles in predicting seizure status (Fig. S3). To complement the classification, we next applied Random Survival Forest under the same cross-validation scheme for survival prediction. Consistent with the classification results, glycomic features alone demonstrated strong discriminative performance in survival prediction, achieving a mean C-index of 0.759, which was comparable to that obtained using cancer grade alone (C-index = 0.816) (Fig. S4A and Table S5). These findings further support the prognostic relevance of glycomics in glioma. Interestingly, Glycan 1996 still stands as the top feature (Fig. S5). To demonstrate the model robustness, bootstrap optimism-corrected C-index estimates showed limited performance inflation (Fig. S4B).

### Survival analysis based on glycomic features

**Fig. 5.**
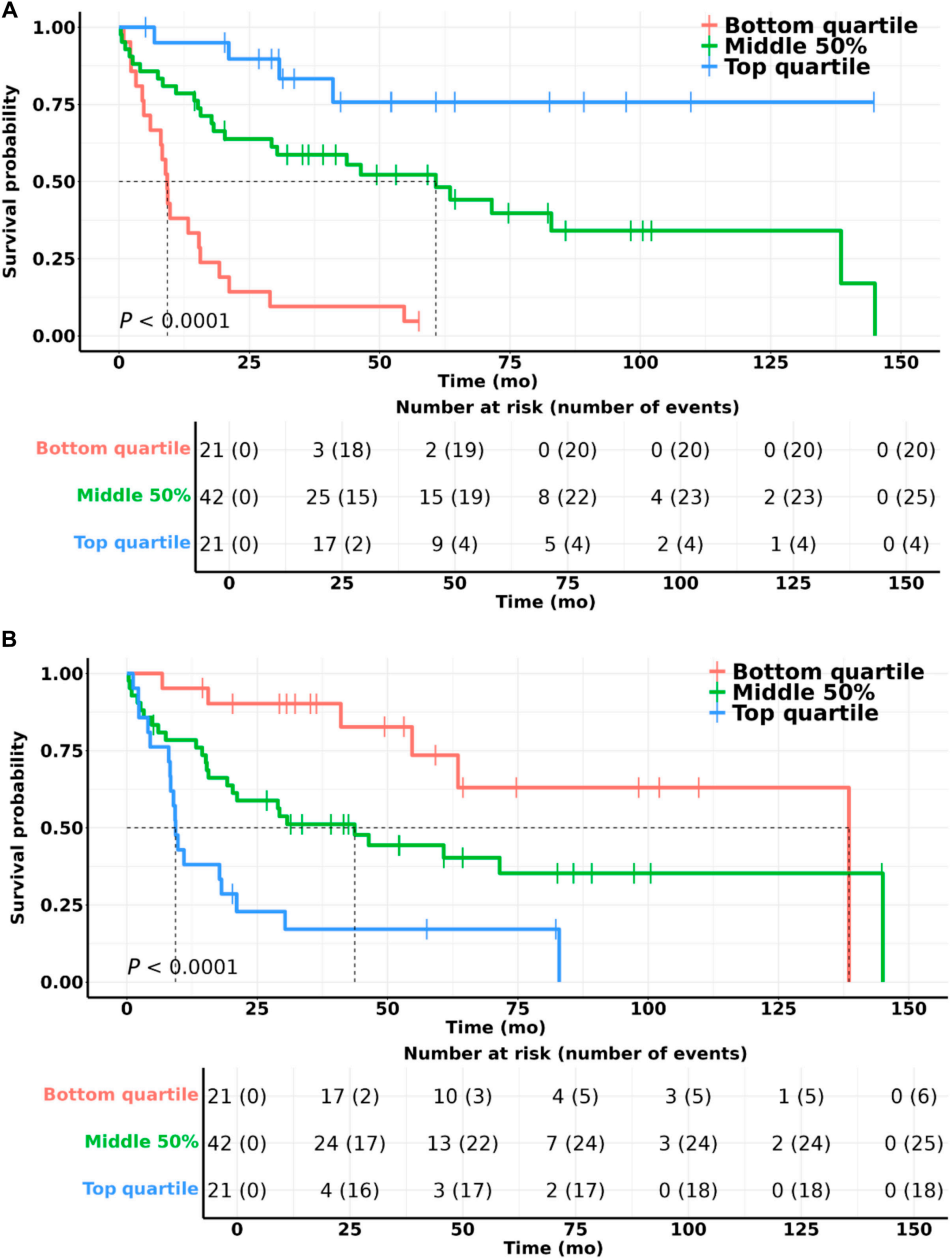
KM survival analyses to assess the impact of 2 informative glycans on survival probability. (A) Stratified by quartiles of Glycan 1996 abundance. (B) Stratified by quartiles of Glycan 1663 abundance.

To further elucidate the relationship between specific glycomic features and patient survival, we conducted KM survival analyses based on the abundance levels of the most predictive glycans identified by the RF model. Patients were stratified into quartiles based on glycan abundance, allowing us to assess the impact of glycan abundance on survival probabilities. For Glycan 1996, which emerged as one of the top predictors in our model, we observed a significant difference in survival outcomes between patients in the lowest and highest quartiles (Fig. 5A). Specifically, patients in the lowest quartile of Glycan 1996 abundance had markedly lower survival probabilities compared to those in the middle and highest quartile (*P* < 0.0001). These data suggest that higher levels of Glycan 1996 are associated with improved survival in patients with glioma, indicating its potential role as a favorable prognostic biomarker. Conversely, for Glycan 1663, the survival trend was reversed. Patients in the highest quartile of Glycan 1663 abundance exhibited significantly lower survival probabilities than those in the lowest quartile (*P* < 0.0001), as shown in Fig. 5B. Thus, elevated levels of Glycan 1663 are associated with poorer survival outcomes, highlighting its potential as an unfavorable prognostic marker. These findings underscore the prognostic significance of specific glycan structures in gliomas. The opposing survival trends observed for Glycan 1996 and Glycan 1663 suggest that different glycans may play distinct roles in tumor biology and progression. High expression of certain glycans may contribute to tumor aggressiveness and reduced patient survival, while others may be indicative of the opposite direction.

## Discussion

In this study, we explored the prognostic potential of glycomic profiling in gliomas to predict patient outcomes using machine-learning approaches. By constructing a TMA from patients with glioma and controls, we enabled a systematic analysis for linking clinical variables and glycomic features to tumor aggressiveness. Initial survival analyses confirmed that seizure and mortality status are significantly associated with cancer grade, though in opposite directions. Higher cancer grades correspond to lower survival rates, whereas the presence of seizure status is associated with better survival outcomes. Moreover, our results indicate that specific glycan structures, such as high-mannose, core-fucosylated, and complex bisecting N-glycans, are associated with cancer grade and may serve as novel biomarkers for gliomas progression. These results underscore the importance of glycomic alterations in glioma biology and their potential role in refining prognostic models.

We selected RF as the prediction model for both mortality and seizure status based on clinical variables, including cancer grade, gender, and age at surgery. To evaluate the potential role of glycomic features as prognostic biomarkers, we trained the RF model with glycomic features alone and compared its performance with the model trained using cancer grade alone, adjusted for gender and age at surgery. We found that glycomic features alone achieved strong predictive performance, with both AUROC and AUPRC value exceeding 0.8, and showed comparable predictive power to cancer grade. Consistent with these findings, survival prediction using Random Survival Forest further demonstrated that glycomic features achieved strong performance in predicting time-to-event outcomes. These findings suggest that glycomic profiling provides prognostic information comparable to key clinical metrics, such as cancer grade, highlighting glycans as an underexplored yet valuable biomarker class in gliomas. Moreover, the success of the RF model using glycomic and clinical data highlights the power of machine learning in disease prognosis and biomarker discovery, particularly for complex diseases like gliomas. For example, Glycan 1996 and Glycan 1663 emerged as important predictors of survival, with contrasting effects on patient outcomes. This indicates that glycomic alterations in gliomas may be multifaceted, contributing both to tumor suppression and progression depending on the specific glycan structure. These findings provide new insights into the role of glycosylation in gliomas and pave the way for further research into how these molecular features can be leveraged to improve patient stratification, potentially aiding in clinical decision-making.

This study has several limitations that need to be addressed in future research. First, the absence of spatial context and cell type composition in our glycomic analysis limits our ability to capture the heterogeneity within the tumor microenvironment, which plays a critical role in glioma progression. Incorporating single-cell resolution spatial glycomics or other spatially resolved omics approaches would provide valuable insights into the localization and function of glycans in relation to tumor architecture. Additionally, the lack of genetic mutation data, such as *IDH1* mutation or *MGMT* promoter methylation status, restricts the comprehensiveness of our prognostic model. These mutations are well known to influence glioma outcomes and should be integrated with glycomic data to build more robust predictive models. Moreover, our sample size is limited to a cohort of patients with glioma from a single region (Kentucky), which may reduce the generalizability of our findings to broader populations. Expanding the cohort to include more diverse patient populations would strengthen the validity of the results and improve the model’s applicability across different demographics. Furthermore, future studies should aim to integrate additional omics modalities, such as genomics, proteomics, and transcriptomics, to develop a more comprehensive and multidimensional approach to gliomas biomarker discovery and prognosis prediction. Lastly, we plan to adopt a more advanced machine-learning approach, such as deep-learning models, to enhance the prediction when the sample collection increases. Multimodal deep-learning models provide such flexibility to incorporate multiple omics modalities as input and model their interactions. Deep learning can also leverage the histology images, which is a crucial component in cancer research, as the training features. By addressing these limitations, future research can further enhance the predictive power and clinical relevance of glycomic biomarkers in gliomas.

## References

[1] Wu W, Klockow JL, Zhang M, Lafortune F, Chang E, Jin L, Wu Y, Daldrup-Link HE. Glioblastoma multiforme (GBM): An overview of current therapies and mechanisms of resistance. Pharmacol Res. 2021;171: Article 105780. 34302977 10.1016/j.phrs.2021.105780 PMC8384724

[2] Abou Jaoude D, Moore JA, Moore MB, Twumasi-Ankrah P, Ablah E, Moore DF Jr. Glioblastoma and increased survival with longer chemotherapy duration. Kans J Med. 2019;12(3):65–69. 31489102 PMC6710024

[3] Crespo I, Vital AL, Gonzalez-Tablas M, del Carmen Patino M, Otero A, Lopes MC, de Oliveira C, Domingues P, Orfao A, Tabernero MD. Molecular and genomic alterations in glioblastoma multiforme. Am J Pathol. 2015;185(7):1820–1833. 25976245 10.1016/j.ajpath.2015.02.023

[4] Molenaar RJ, Verbaan D, Lamba S, Zanon C, Jeuken JW, Boots-Sprenger SH, Wesseling P, Hulsebos TJ, Troost D, van Tilborg AA. et al. The combination of IDH1 mutations and MGMT methylation status predicts survival in glioblastoma better than either IDH1 or MGMT alone. Neuro-Oncology. 2014;16(9):1263–1273. 24510240 10.1093/neuonc/nou005 PMC4136888

[5] Kourou K, Exarchos TP, Exarchos KP, Karamouzis MV, Fotiadis DI. Machine learning applications in cancer prognosis and prediction. Comput Struct Biotechnol J. 2015;13:8–17. 25750696 10.1016/j.csbj.2014.11.005 PMC4348437

[6] Rouzbahani AK, Khalili-Tanha G, Rajabloo Y, Khojasteh-Leylakoohi F, Garjan HS, Nazari E, Avan A. Machine learning algorithms and biomarkers identification for pancreatic cancer diagnosis using multi-omics data integration. Pathol Res Pract. 2024;263: Article 155602. 39357184 10.1016/j.prp.2024.155602

[7] Zhang B, Shi H, Wang H. Machine learning and AI in cancer prognosis, prediction, and treatment selection: A critical approach. J Multidiscip Healthc. 2023;16:1779–1791. 37398894 10.2147/JMDH.S410301 PMC10312208

[8] Khalighi S, Reddy K, Midya A, Pandav KB, Madabhushi A, Abedalthagafi M. Artificial intelligence in neuro-oncology: Advances and challenges in brain tumor diagnosis, prognosis, and precision treatment. NPJ Precis Oncol. 2024;8(1):80. 38553633 10.1038/s41698-024-00575-0 PMC10980741

[9] Babaei Rikan S, Sorayaie Azar A, Naemi A, Bagherzadeh Mohasefi J, Pirnejad H, Wiil UK. Survival prediction of glioblastoma patients using modern deep learning and machine learning techniques. Sci Rep. 2024;14(1):2371. 38287149 10.1038/s41598-024-53006-2 PMC10824760

[10] Guo J, Fathi Kazerooni A, Toorens E, Akbari H, Yu F, Sako C, Mamourian E, Shinohara RT, Koumenis C, Bagley SJ, et al. Integrating imaging and genomic data for the discovery of distinct glioblastoma subtypes: A joint learning approach. Sci Rep. 2024;14(1):4922. 38418494 10.1038/s41598-024-55072-y PMC10902376

[11] Veillon L, Fakih C, Abou-El-Hassan H, Kobeissy F, Mechref Y. Glycosylation changes in brain cancer. ACS Chem Neurosci. 2018;9(1):51–72. 28982002 10.1021/acschemneuro.7b00271 PMC5771830

[12] Sun YF, Zhang LC, Niu RZ, Chen L, Xia QJ, Xiong LL, Wang TH. Predictive potentials of glycosylation-related genes in glioma prognosis and their correlation with immune infiltration. Sci Rep. 2024;14(1):4478. 38396140 10.1038/s41598-024-51973-0 PMC10891078

[13] Demirhan DB, Yilmaz H, Erol H, Kayili HM, Salih B. Prediction of gastric cancer by machine learning integrated with mass spectrometry-based N-glycomics. Analyst. 2023;148:2073–2080. 37009642 10.1039/d2an02057b

[14] Vathy-Fogarassy A, Gombas V, Torok R, Jarvas G, Guttman A. Improved analytical workflow towards machine learning supported N-glycomics-based biomarker discovery. Talanta. 2025;295: Article 128389. 40449373 10.1016/j.talanta.2025.128389

[15] Zhang H, Liu S, Wang Y, Huang H, Sun L, Yuan Y, Cheng L, Liu X, Ning K. Deep learning enhanced the diagnostic merit of serum glycome for multiple cancers. iScience. 2024;27(1): Article 108715. 38226168 10.1016/j.isci.2023.108715 PMC10788220

[16] Conroy LR, Clarke HA, Allison DB, Valenca SS, Sun Q, Hawkinson TR, Young LE, Ferreira JE, Hammonds AV, Dunne JB, et al. Spatial metabolomics reveals glycogen as an actionable target for pulmonary fibrosis. Nat Commun. 2023;14(1):2759. 37179348 10.1038/s41467-023-38437-1 PMC10182559

[17] Sun RC, Young LE, Bruntz RC, Markussen KH, Zhou Z, Conroy LR, Hawkinson TR, Clarke HA, Stanback AE, Macedo JK, et al. Brain glycogen serves as a critical glucosamine cache required for protein glycosylation. Cell Metab. 2021;33(7):1404–1417.e9. 34043942 10.1016/j.cmet.2021.05.003 PMC8266748

[18] Conroy LR, Stanback AE, Young LE, Clarke HA, Austin GL, Liu J, Allison DB, Sun RC. In situ analysis of N-linked glycans as potential biomarkers of clinical course in human prostate cancer. Mol Cancer Res. 2021;19(10):1727–1738. 34131069 10.1158/1541-7786.MCR-20-0967 PMC8492534

[19] Stanback AE, Conroy LR, Young LE, Hawkinson TR, Markussen KH, Clarke HA, Allison DB, Sun RC. Regional N-glycan and lipid analysis from tissues using MALDI-mass spectrometry imaging. STAR Protoc. 2021;2(1): Article 100304. 33554139 10.1016/j.xpro.2021.100304 PMC7848795

[20] Clarke HA, Ma X, Shedlock CJ, Medina T, Hawkinson TR, Wu L, Ribas RA, Keohane S, Ravi S, Bizon JL, et al. Spatial mapping of the brain metabolome lipidome and glycome. Nat Commun. 2025;16(1):4373. 40355410 10.1038/s41467-025-59487-7 PMC12069719

[21] Clarke HA, Hawkinson TR, Shedlock CJ, Medina T, Ribas RA, Wu L, Liu Z, Ma X, Xia Y, Huang Y, et al. Glycogen drives tumour initiation and progression in lung adenocarcinoma. Nat Metab. 2025;7(5):952–965. 40069440 10.1038/s42255-025-01243-8 PMC12116239

[22] Fan X, Li Y, Shan X, You G, Wu Z, Li Z, Qiao H, Jiang T. Seizures at presentation are correlated with better survival outcomes in adult diffuse glioma: A systematic review and meta-analysis. Seizure. 2018;59:16–23. 29727741 10.1016/j.seizure.2018.04.018

[23] Singh S, Mehrotra A, Kanjilal S, Paliwal VK. Effect of seizure timing on long-term survival in patients with brain tumor. Epilepsy Behav. 2021;117: Article 107566. 10.1016/j.yebeh.2020.107566 33610463

