# Supplementary material for: Machine-Learning-Based Prediction of Clinical Outcomes in Gliomas Using Glycomic Features
Source: Comput Struct Biotechnol J. 2026 Jul 9;35(1):0153. doi: 10.34133/csbj.0153 (PMC13346664; doi:10.34133/csbj.0153)
Supplement: Supplementary 1 — Figs. S1 to S5 Tables S1 to S5 [file csbj.0153.f1.docx]

**Supplementary materials for** **machine learning-based prediction of clinical outcomes in gliomas using glycomics features**

**Supplementary Tables**

| **Model** | **n** | **AUROC**  **mean** | **AUROC**  **sd** | **AUROC**  **low** | **AUROC**  **high** | **AUPRC**  **mean** | **AUPRC**  **sd** | **AUPRC**  **low** | **AUPRC**  **high** |
| --- | --- | --- | --- | --- | --- | --- | --- | --- | --- |
| Lasso | 50 | 0.894 | 0.083 | 0.871 | 0.917 | 0.933 | 0.068 | 0.914 | 0.952 |
| RF | 50 | 0.928 | 0.066 | 0.910 | 0.946 | 0.957 | 0.049 | 0.944 | 0.971 |
| SVM | 50 | 0.906 | 0.075 | 0.885 | 0.927 | 0.936 | 0.084 | 0.912 | 0.959 |

**Table S1. Summary of mortality status prediction results of different models across five-fold cross-validation with 10 repetitions (n = 50).**

| **Feature** | **n** | **AUROC**  **mean** | **AUROC**  **sd** | **AUROC**  **low** | **AUROC**  **high** | **AUPRC**  **mean** | **AUPRC**  **sd** | **AUPRC**  **low** | **AUPRC**  **high** |
| --- | --- | --- | --- | --- | --- | --- | --- | --- | --- |
| Cancer Grade | 50 | 0.929 | 0.065 | 0.911 | 0.947 | 0.958 | 0.048 | 0.945 | 0.971 |
| Glycomics | 50 | 0.880 | 0.072 | 0.860 | 0.900 | 0.925 | 0.055 | 0.910 | 0.941 |

**Table S2. Summary of mortality status prediction results using different feature sets (clinical vs glycomics) across five-fold cross-validation with 10 repetitions (n = 50).**

| **Model** | **n** | **AUROC**  **mean** | **AUROC**  **sd** | **AUROC**  **low** | **AUROC**  **high** | **AUPRC**  **mean** | **AUPRC**  **sd** | **AUPRC**  **low** | **AUPRC**  **high** |
| --- | --- | --- | --- | --- | --- | --- | --- | --- | --- |
| Lasso | 50 | 0.748 | 0.115 | 0.717 | 0.780 | 0.747 | 0.136 | 0.709 | 0.785 |
| RF | 50 | 0.754 | 0.118 | 0.722 | 0.787 | 0.750 | 0.130 | 0.714 | 0.786 |
| SVM | 50 | 0.722 | 0.138 | 0.684 | 0.760 | 0.735 | 0.152 | 0.693 | 0.778 |

**Table S3. Summary of seizure status prediction results of different models across five-fold cross-validation with 10 repetitions (n = 50).**

| **Feature** | **n** | **AUROC**  **mean** | **AUROC**  **sd** | **AUROC**  **low** | **AUROC**  **high** | **AUPRC**  **mean** | **AUPRC**  **sd** | **AUPRC**  **low** | **AUPRC**  **high** |
| --- | --- | --- | --- | --- | --- | --- | --- | --- | --- |
| Cancer Grade | 50 | 0.756 | 0.116 | 0.724 | 0.788 | 0.754 | 0.126 | 0.719 | 0.789 |
| Glycomics | 50 | 0.778 | 0.110 | 0.747 | 0.808 | 0.766 | 0.141 | 0.727 | 0.806 |

**Table S4. Summary of seizure status prediction results using different feature sets (clinical vs glycomics) across five-fold cross-validation with 10 repetitions (n = 50).**

| **Model** | **Cindex**  **mean** | **Cindex**  **sd** | **Cindex**  **Pooled** | **CI_Lower** | **CI_Upper** | **Apparent** | **Optimism** | **Corrected** |
| --- | --- | --- | --- | --- | --- | --- | --- | --- |
| Cancer Grade | 0.816 | 0.060 | 0.777 | 0.759 | 0.795 | 0.813 | 0.004 | 0.809 |
| Glycomics | 0.759 | 0.084 | 0.736 | 0.713 | 0.758 | 0.808 | 0.034 | 0.774 |

**Table S5. Summary of time-to-event survival outcome prediction results using different feature sets (clinical vs glycomics) across five-fold cross-validation with 10 repetitions (n = 50**).

**Supplementary Figures**


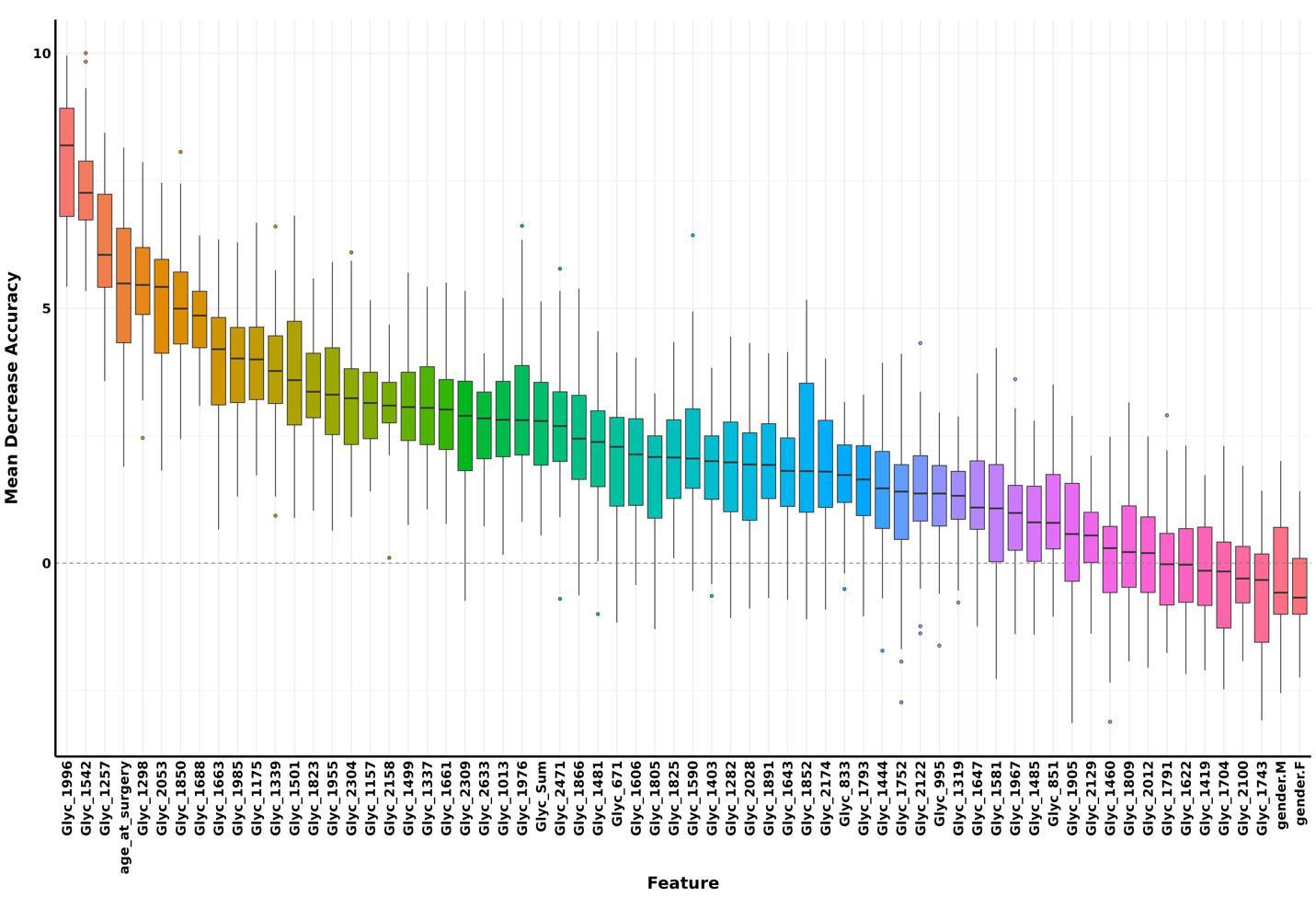


**Figure S1.** Feature importance measured by permutation importance from random forest model for predicting mortality status using glycomic features, adjusted for gender and age at surgery.


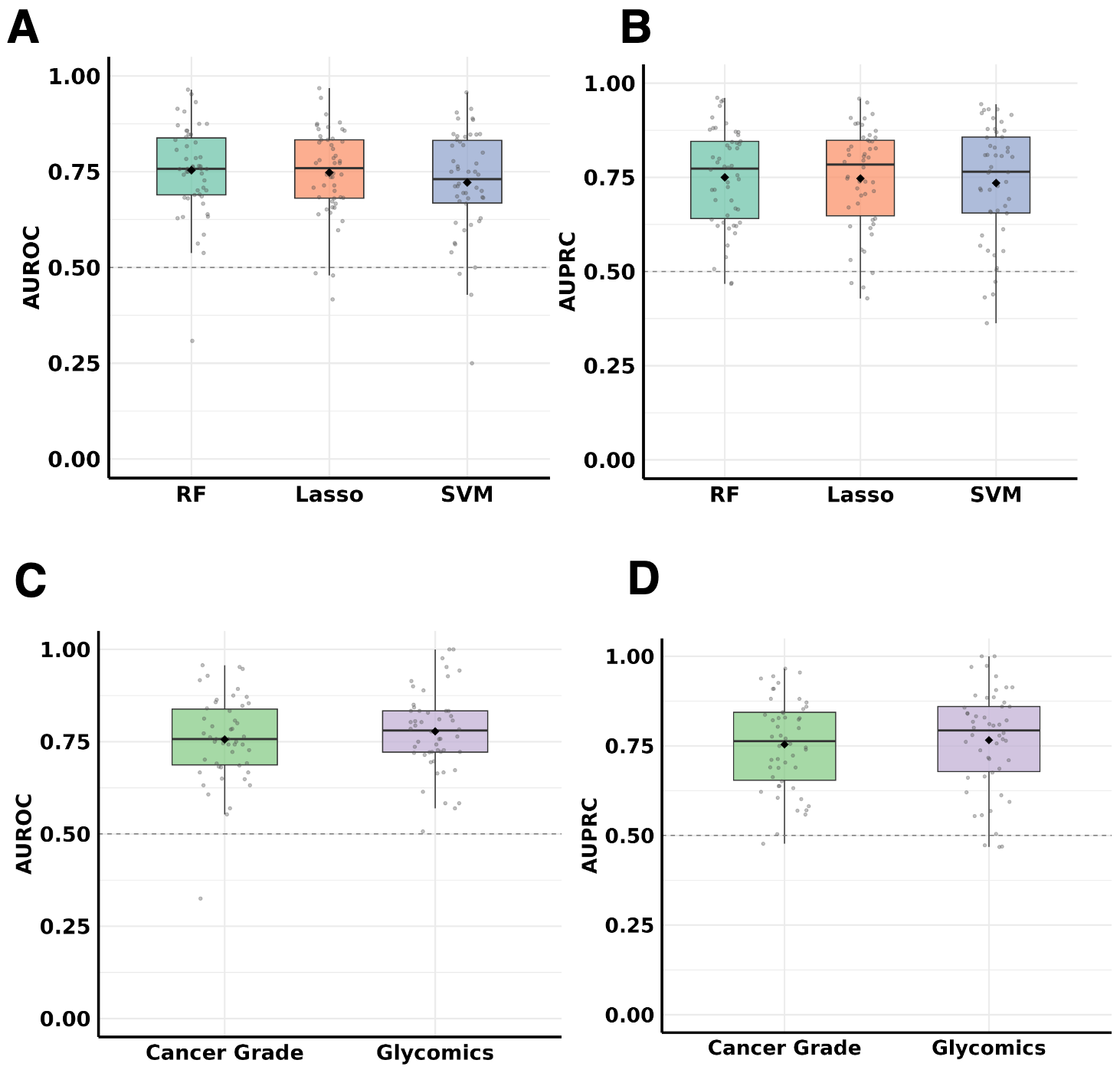


**Figure S2.** Predicting the seizure status by cancer grade or glycomic features. **(A)** AUROC reported by three machine learning models solely using cancer grade, adjusted for gender and age at surgery **(B)** AUPRC reported by three machine learning models solely using cancer grade, adjusted for gender and age at surgery **(C)** AUROC reported by Random Forest using cancer grade or glycomics, adjusted for gender and age at surgery **(D)** AUPRC reported by Random Forest using cancer grade or glycomics, adjusted for gender and age at surgery.


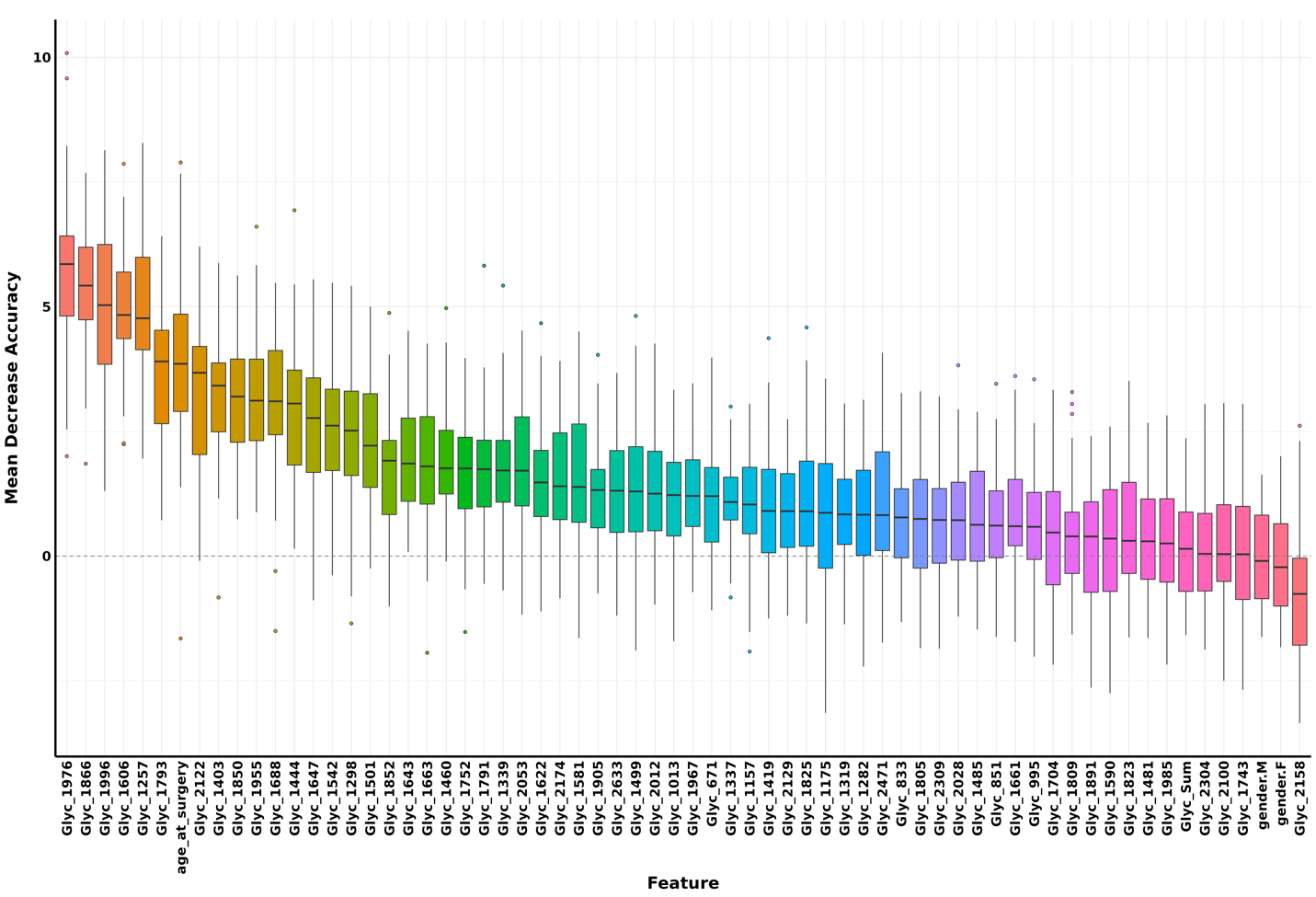


**Figure S3.** Feature importance measured by permutation importance from Random Forest model for predicting seizure status using glycomic features, adjusted for gender and age at surgery.


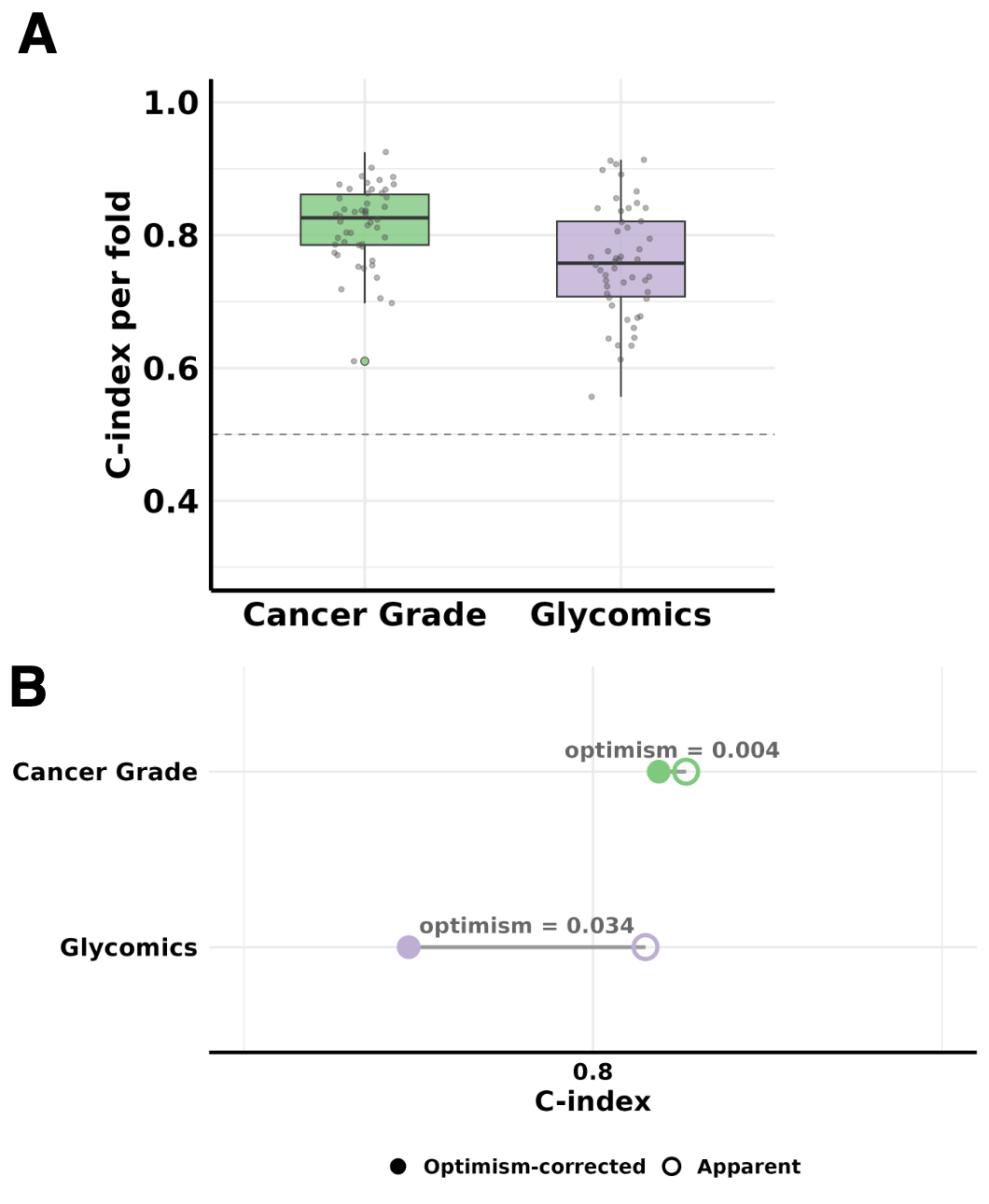


**Figure S4.** Survival prediction by cancer grade or glycomic features. **(A)** C-index reported by Random Survival Forest using cancer grade and glycomics, adjusted for gender and age at surgery **(B)** C-index reported by the Random Survival Forest model with optimism correction.


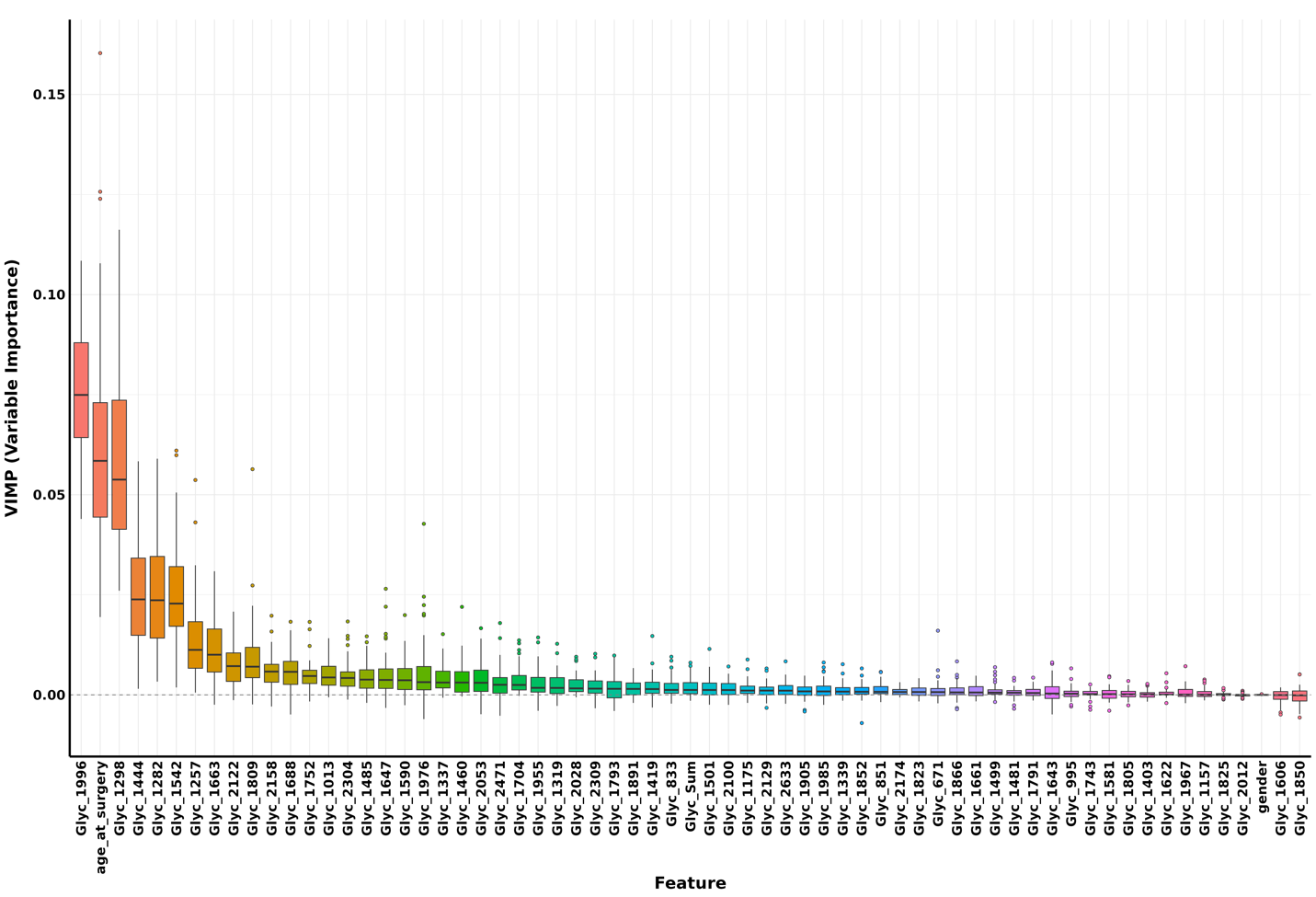


**Figure S5.** Feature importance measured by permutation importance from Random Survival Forest model for survival prediction using glyomic features, adjusted for gender and age at surgery.
